# Conjugating time and frequency: hemispheric specialization, acoustic uncertainty, and the mustached bat

**DOI:** 10.3389/fnins.2015.00143

**Published:** 2015-04-27

**Authors:** Stuart D. Washington, John S. Tillinghast

**Affiliations:** ^1^Center for Functional and Molecular Imaging, Georgetown University Medical CenterWashington, DC, USA; ^2^Department of Neurology, Georgetown University Medical CenterWashington, DC, USA; ^3^Center for Neuroscience Research, Children's National Medical CenterWashington, DC, USA; ^4^Department of Mathematics and Statistics, American UniversityWashington, DC, USA; ^5^Department of Statistics, The George Washington UniversityWashington, DC, USA

**Keywords:** acoustic uncertainty, echolocation, Heisenberg-Gabor limit, hemispheric specialization, mustached bats, music, speech, spectral-temporal

## Abstract

A prominent hypothesis of hemispheric specialization for human speech and music states that the left and right auditory cortices (ACs) are respectively specialized for precise calculation of two canonically-conjugate variables: time and frequency. This spectral-temporal asymmetry does not account for sex, brain-volume, or handedness, and is in opposition to closed-system hypotheses that restrict this asymmetry to humans. Mustached bats have smaller brains, but greater ethological pressures to develop such a spectral-temporal asymmetry, than humans. Using the Heisenberg-Gabor Limit (i.e., the mathematical basis of the spectral-temporal asymmetry) to frame mustached bat literature, we show that recent findings in bat AC (1) support the notion that hemispheric specialization for speech and music is based on hemispheric differences in temporal and spectral resolution, (2) discredit closed-system, handedness, and brain-volume theories, (3) underscore the importance of sex differences, and (4) provide new avenues for phonological research.

## Hemispheric lateralization for language: a multi-faceted controversy

The finding that damage to the left cerebral hemisphere in humans impairs receptive language (e.g., speech perception) is seminal to the field of neuroscience (Wernicke, 1874). More precisely, damage to portions of the temporal lobe in a human's left cerebral hemisphere disrupts one's ability to comprehend vocalizations that symbolize objects, ideas, and meanings to oneself and other humans (e.g., words, phrases, and sentences). Comparable right hemispheric damage has fewer effects on receptive language but impairs musical processing (Milner, 1962; Samson and Zatorre, 1991; Zatorre et al., 1994) and pitch discrimination (Sidtis and Volpe, 1988; Robin et al., 1990; Zatorre et al., 1994) as well as the ability to identify a speaker and the prosody of his or her speech (Robinson and Fallside, 1991). Both classical and modern studies commonly show that left cerebral specialization for receptive language is less pronounced in human females than in conspecific males (Lansdell, 1964; McGlone, 1977; Shaywitz et al., 1995).

Neuroscientists have proposed numerous hypotheses to explain this asymmetry. An early hypothesis erroneously credited to Paul Broca relates both left lateralization of language function and, via decussation, right handedness to a general left hemispheric dominance common to most humans (Harris, 1991). This “Broca handedness rule” implies that *most* left handed people display right hemispheric dominance for language, an assertion not validated by rigorous empirical studies (Knecht et al., 2000). However, since human tool usage is irrefutably the most advanced in the animal kingdom and is inexorably linked to handedness, the “Broca handedness rule” is appealing as it links left-lateralized cortical control of handedness to left-lateralized cortical control of speech and language. Another hypothesis proposes that in larger mammalian brains, such as those of humans, time-critical neuronal computations strain the capacity of the corpus callosum and would be performed more quickly by intrahemispheric circuits (Ringo et al., 1994). The *brain-volume hypothesis* implies that hemispheric specialization for communication sound processing would be greater in the left hemispheres of mammals with greater brain volumes than humans, such as proboscidea (e.g., elephants) and cetaceans (e.g., dolphins). These two hypotheses are generally respected as plausible explanations for language lateralization in humans and are not mutually exclusive.

Two other hypotheses are mutually exclusive and have thus generated much debate in the last half century. The first of these is the “closed system” hypothesis, which argues that neural mechanisms underlying receptive auditory communication in humans (i.e., speech perception) are unique to humans, specific for speech and language, and are contained within a “speech organ” in the left hemisphere (Liberman and Mattingly, 1989). Specifically, advocates of the “closed system” hypothesis state that neurons comprising this uniquely human, left-lateralized “speech organ” exclusively process linguistically salient aspects of speech sounds, such as consonants and vowels, and relegate the processing of pitch, loudness, timbre, and location to other, less specialized auditory neural substrates.

The various “domain-general” hypotheses, which state that speech sounds are processed by the same neural substrates as all other sounds and describe language dominance via auditory signal processing, are irreconcilable with the “closed system” hypothesis. Though domain-general hypotheses differ, most implicate a fundamental principle of acoustics called “acoustic uncertainty” as the underlying, evolutionary force driving left hemispheric dominance for language (Zatorre et al., 2002). The acoustic uncertainty principle describes a trade-off between time and frequency such that, at the upper limit of resolution, increasing time (temporal) resolution can only be achieved at the expense of frequency (spectral) resolution and vice-versa. Domain-general theories emerged from decades of observations showing temporal domain processing deficits across multiple language disorders, including aphasia (Efron, 1963; Tallal and Piercy, 1973, 1975), dyslexia (Tallal, 1980; Tallal et al., 1993; Temple et al., 2000), and dysphasia (Tallal and Newcombe, 1978; Tallal et al., 1991, 1993). These language studies were followed by other studies showing either deficits in the spectral domain following right hemispheric lesions (Zatorre, 1985, 1988; Samson and Zatorre, 1988) or a double-dissociation for temporal or spectral domain processing following left or right hemispheric lesions, respectively (Robin et al., 1990). Advocates of domain-general hypotheses argue that both the left and right auditory cortices process speech and other sounds, but only the left auditory cortex has the temporal resolution necessary to differentiate consonant sounds by their formant transition rates and voice-onset-times. A lack of such temporal resolution, either by congenital defect or neurological damage, would render most consonant sounds indistinguishable and thus most spoken languages incomprehensible. In what would appear to be a classic example of “multiple independent discovery,” two research groups proposed similar domain-general models based around hemispheric differences in spectral and temporal resolution at nearly the same time (Zatorre et al., 2002; Poeppel, 2003). To avoid favoring one set of terminology over the other, we will use the acronym *Asymmetry for Spectral versus Temporal Integration and Resolution* (ASTIR) as an umbrella-term for hypotheses that explain auditory hemispheric specialization via a trade-off between acoustic spectral and temporal resolution (see Supplementary Section 1 for a more in depth perspective). Each of the hypotheses conforming to ASTIR shares one polarizing implication: hemispheric specialization should emerge within the brains of any species whose survival hinges upon extracting refined temporal and spectral information from the auditory signals in its environment regardless of brain volume or handedness.

Our aim here is to further validate either the closed-system or domain-general hypothesis of receptive auditory communication by exploring this last implication of ASTIR. We base our exploration of ASTIR around the functional organization of the mustached bat (*Pteronotus parnellii*) auditory cortex due to (1) the fact that individuals in this species primarily orient themselves and communicate with each other using complex auditory signals and (2) the vast and well-established literature describing the auditory cortical maps in this species.

## The heisenberg-gabor limit: the mathematical basis for acoustic uncertainty

*The Acoustic Uncertainty Principle* is one of the many uncertainty principles common to physical sciences. Uncertainty principles are defined by mathematical inequalities that place a limit on the precision of simultaneous measurements of two canonically-conjugate variables (i.e., variables that are Fourier transformations of one another) (Joos, 1948). *The Acoustic Uncertainty Principle* states that frequency and time are canonically-conjugate variables of sound waves. The mathematical basis of acoustic uncertainty is the Heisenberg-Gabor Limit (Schuller and Batliner, 2014), a principle of signal processing that is applicable to all functions and which states:

$$\Delta f\cdot \Delta t\geq \frac{1}{4\pi },$$

where Δ*f* is the standard deviation of frequency and Δ*t* is the standard deviation of time from the peak intensity of the signal.

**Figure 1:**
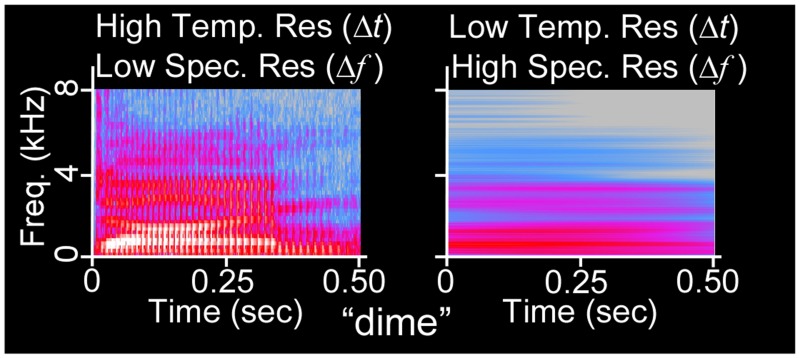
**Acoustic uncertainty as illustrated via spectrogram**. The waveform of the 500 ms duration word “dime” (NU-6 List 1A) has been spectrographically analyzed using Fourier transforms with (left) narrow temporal integration windows (256 frequency channels, Hanning window) and (right) wide temporal integration windows (32768 frequency channels). At left, *glottal pulses* (vertical lines) and *formant transitions* (changes in vocal spectral peaks over time) are visible, whereas harmonics are not. At right, harmonics are visible, but the sequence of auditory events is confounded.

The Heisenberg-Gabor Limit demonstrates that simultaneous, precise measurements of a function in both the temporal (time) and spectral (frequency) domains is impossible, because refined temporal resolution only comes at the expense of spectral resolution and vice-versa, as shown in Figure 1. In terms of short-time Fourier transformations, a wide temporal window permits refined spectral resolution whereas a narrow temporal window permits refined temporal resolution. Such a multi-window system by definition resigns itself to measuring spectral and temporal components of the same sound on different time scales. We mathematically articulate similarities between the Heisenberg's Quantum Uncertainty Principle and the Acoustic Uncertainty Principle and further explore both concepts in Supplementary Section 1.

Independent groups of researchers postulated that the specialization for speech and music characteristic of the left and right hemispheres of humans, respectively, stems from the use of narrow temporal windows by left auditory cortex and wide temporal windows by right auditory cortex (Zatorre et al., 2002; Poeppel, 2003). This cortical asymmetry may stem from a right ear advantage for temporal information and a left ear advantage for spectral information that is evident at the level of the cochlea (Sininger and Cone-Wesson, 2004), a natural spectrum analyzer also subject to the Heisenberg-Gabor Limit. The open-ended mathematical nature of ASTIR suggests that such hemispheric specialization would develop in any mammalian species whose survival hinges upon the extraction of precise spectral and temporal information from sounds. For instance, acquisition of both refined temporal and spectral information is key to the survival of mustached bats.

## Echolocation in mustached bats: new perspectives on a classic model

**Figure 2:**
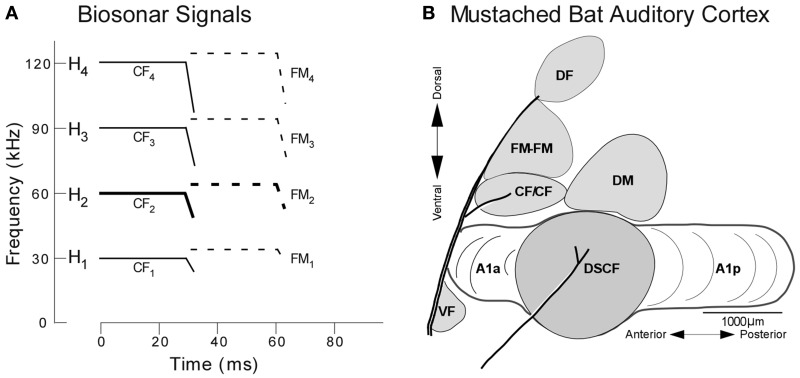
**Echolocation and the functional organization of the mustached bat auditory cortex. (A)** H_1−4_ refer to harmonics 1 through 4 of the echolocation pulse or echo. Note the constant frequency (CF) and frequency-modulated (FM) components present in the pulse and echo. **(B)** Lateral view of the mustached bat auditory cortex showing the location of the DSCF area (shown in gray) as defined based on its role in computing biosonar signals. Anatomical landmarks (blood vessels shown by thick lines) and tuning properties of neuronal responses were used to identify the Doppler-shifted constant frequency (DSCF), anterior primary auditory (A1a), posterior primary auditory (A1p), dorsomedial (DM), CF/CF, FM-FM, and dorsal fringe (DF) areas (adapted from Suga et al., 1983).

The behavior of mustached bats and the functional organization of their auditory cortices has been explored primarily from the perspective of *echolocation*, the method by which micro-bats (microchiroptera) generate sonar signals to orient themselves and hunt insects (Suga, 1985). During echolocation, mustached bats emit sounds that are comprised of a constant frequency (CF) and downward frequency modulation (FM) and the three harmonics thereof. These four signals (fundamental + 3 harmonics) are labeled H_1−4_, where the fundamental and each harmonic are composed of CF (CF_1−4_) and FM (FM_1−4_) components (Figure 2). When flying toward a stationary target, a bat detects both its pulse (i.e., emitted) and echo (i.e., returning) signals, the latter of which has been Doppler-shifted upward in frequency relative to the former. Any possibility of masking by temporal overlap between the pulse and echo is averted since the mustached bat's auditory periphery evolved an enhanced sensitivity to the echo-CF_2_ (60–63 kHz in *P.p. parnellii*, Kanwal et al., 1999, and 57.5–60 kHz in *P.p. rubiginosus*, Xiao and Suga, 2002) and a relative insensitivity to the pulse-CF_2_ (Suga, 1985; Kanwal, 1999; Kanwal et al., 1999).

Doppler-shifts and echo-delays respectively impose key spectral and temporal changes to the echo H_1−4_ that differentiate it from the pulse H_1−4_, and these differences are in turn exploited by “combination-sensitive” neurons in the mustached bat auditory cortex (Suga, 1978). The neural responses of combination-sensitive neurons are *facilitated* when certain CF and FM components of the pulse and echo are presented together, such that the facilitated responses are greater (i.e., a greater spike count or higher spike rate) than the sum of the responses elicited when the individual CF and FM components are presented alone. For instance, neurons in the FM-FM processing subregion are facilitated by pulse-echo pairs of FMs (e.g., pulse-FM_1_+echo-FM_3_) and tuned to their delays (i.e., inter-stimulus-intervals, 0.4–18 ms) (O'Neill and Suga, 1979). The temporal combination sensitivity of FM-FM neurons enables the bat to detect target range, and the spatial organization of FM-FM neurons forms a cortical map of ranges based on echo-delays in non-primary auditory cortex. Thus, the bat is able to receive accurate range information due to the refined temporal processing of neurons in its auditory cortex.

The CF/CF processing area contains neurons that are facilitated when CFs in the pulse-CF_1_ range are combined with CFs in either the echo-CF_2_ or echo-CF_3_ ranges. Similarly, neurons in the Doppler-shifted constant frequency (DSCF) processing subregion (sometimes referred as the *auditory fovea* Schnitzler and Denzinger, 2011) are facilitated when CFs in the echo-CF_2_ range are paired at onset with CFs in the pulse-FM_1_ range (23–27 kHz) (Fitzpatrick et al., 1993; Kanwal et al., 1999). The refined spectral resolution of CF/CF and DSCF neurons enables them to distinguish between the pulse- and echo-CF_2_ or, in the case of some CF/CF neurons, the pulse- and echo-CF_3_. Thus, the bat is able to receive accurate velocity information due to the refined spectral processing of neurons in its auditory cortex. Both the CF/CF and DSCF areas contain maps of relative velocities derived from representations of frequencies at or near echo-CF_2_ or, in the case of some CF/CF neurons, echo-CF_3_ (Suga and Jen, 1976). The CF/CF area and its velocity map occupy a relatively small portion of the bat's auditory cortex. The DSCF area and its velocity map, on the other hand, occupy the center-most 50% of the primary auditory cortex (A1) and will be of particular importance to this discussion going forward.

For mustached bats, both refined spectral and temporal resolution are essential to tracking the velocity and range of targets. Like momentum and position or frequency and time, precise sonar measurements of velocity and range are impossible to achieve on the same time scale (Parker, 2011). This *Doppler Ambiguity* could explain nuances of the mustached bat's echolocation behavior. As Figure 2 shows, when the bat is at rest, the 20-ms CF components of biosonar signals always precede the 3-ms FM components (Suga, 1985). Thus, the bat processes any spectral differences between the pulse and echo CF components imposed by Doppler-shifts (i.e., target velocity) via a wide temporal window prior to processing temporal differences (delays) between the pulse and echo FM in a narrower window. We detail the role of acoustic uncertainty in the context of mustached bat pursuit behavior in Supplementary Section 2. Pharmacobehavioral results confirm that (1) mustached bats can discriminate between 20-ms CFs presented within the pulse- and echo-CF_2_ ranges with a 0.05 kHz resolution and (2) this refined frequency discrimination is performed by neurons in the DSCF area (Riquimaroux et al., 1992). Substituting Δt in the Heisenberg-Gabor Limit formula with 20 ms shows that the maximum frequency discrimination (i.e., spectral resolution) possible using a typical echo-CF_2_ is 4 Hz (or 0.004 kHz).

Doppler Ambiguity exposes a potential flaw in ASTIR. Velocity and range are processed within the bilateral, cortically adjacent subregions of DSCF, CF/CF, and FM-FM, despite being canonically-conjugate variables. Thus, echolocation demonstrates that the ethological need to precisely calculate canonically-conjugate variables is not necessarily sufficient biological pressure to impose hemispheric specialization. Indeed, anterior auditory field (AAF) in rodents (Linden et al., 2003; Trujillo et al., 2011) and cats (Schreiner and Urbas, 1988; Tian and Rauschecker, 1994; Imaizumi et al., 2004; Carrasco and Lomber, 2009) as well as the AAF homolog of the rhesus macaque (rostral auditory field, or Field R) (Rauschecker et al., 1997) are specialized for faster temporal processing relative to A1. It is conceivable that AAF and A1, like FM-FM and DSCF, could process canonically-conjugate variables like time and frequency bilaterally, making ASTIR unnecessary. Such an issue would be a stronger criticism of ASTIR if the extent of our knowledge on mustached bat auditory cortex were limited to its role in echolocation.

## Conflict and concord: social call and biosonar signal processing in the FM-FM and DSCF areas

Among animals, only human speech (Liberman et al., 1967) and the social calls of cetaceans (Payne and McVay, 1971), mimicking birds (Marler and Pickert, 1984), and some primates (Sutton, 1979) show equal or greater spectrotemporal acoustic complexity than those of mustached bats and other CF-FM bats (Kanwal et al., 1994; Kanwal, 1999; Clement et al., 2006; Ma et al., 2006). Multi-dimensional scaling helped to classify the 19 recurring mustached bat social call syllables as CFs, FMs, or NBs (noisebands) (Kanwal et al., 1994). Unlike the repeating, stereotypic call sequences of frogs (Wells and Schwartz, 1984) and song birds (Marler and Pickert, 1984), mustached bats emit a variety of simple syllabic social calls and calls that are composites of simple syllables. These composite calls reveal a phonetic-like syntax to mustached bat communication, as only 11 of the 19 syllables are even used to construct composites and mustached bats emit only 4% (15/342) of all the possible composites.

Classic studies of biosonar signal processing in mustached bat auditory cortex described the FM-FM, CF/CF, and DSCF areas as “specialized” for echolocation. Prominent language researchers interpreted this specialization for echolocation as a closed-system, likening it to and presenting it as evidence for the closed-system model of speech (Liberman and Mattingly, 1989). CF/CF neural responses to social calls have not been sufficiently studied to warrant discussion here. However, neurons in the FM-FM (Esser et al., 1997; Kanwal, 1999, 2006) and DSCF (Kanwal, 1999, 2006; Washington and Kanwal, 2008) areas respond robustly to conspecific social call syllables, a result noted by critics of closed-system models of speech (Tallal, 2012). Call selectivity within the FM-FM and DSCF areas has a semblance of compatibility with their respective temporal (range) and spectral (velocity) domain processing roles in echolocation. FM-FM neuron responses to composite social calls decline when an artificial silent interval is introduced between the two simple syllables; as the duration of that silent interval increases, FM-FM neuron responses monotonically decrease (Ohlemiller et al., 1994; Esser et al., 1997). Furthermore, either reversing the natural order of composite calls or presenting a simple or composite call in reverse is sufficient to reduce the magnitude of FM-FM neural responses to social calls (Esser et al., 1997). Each of these experimental manipulations had the effect of corrupting the natural temporal structure of social calls and of diminishing excitatory responses of temporally combination sensitive FM-FM neurons.

Likewise, the magnitudes of DSCF neuron responses to certain social calls are known to be comparable to and may even surpass the magnitudes of their responses to pulse-echo CF components (Kanwal, 1999, 2006). Call selectivity in DSCF neurons is based primarily on spectral facilitation. Specifically, when the spectral components of social calls that traverse both the pulse-FM_1_ and echo-CF_2_ ranges are extracted from the call and presented separately, the neuron's response to both call-components (pulse-FM_1_-range+echo-CF_2_-range) is facilitated such that its magnitude is greater than the sum of response magnitudes elicited by each call-component alone. Such band-pass filtered call-components may elicit responses of greater magnitude than the entire natural social call due to the absence of spectral energy traversing inhibitory response areas. Further, similar to FM-FM neurons, DSCF neuron responses to social calls are greatly diminished by reversing the call, but this phenomenon in DSCF neurons may be attributed to asymmetrical inhibitory areas flanking the narrow, excitatory echo-CF_2_ range.

Temporal processing of social calls amongst neurons in the FM-FM area appears concordant with their role in calculating target range during echolocation. However, the means by which DSCF neurons process social calls often differs from how they calculate target velocity via Doppler-shift. Most simple call syllables of mustached bats contain linear, curvilinear, or sinusoidal FMs. Frequency-modulated mustached bat social calls often contain FMs with rates surpassing 500 Hz/ms (e.g., bent-upward FM), and some curvilinear or sinusoidal FM calls have instantaneous rates higher than 5 kHz/ms (e.g., stretched-rippled FM and checked-downward FM). Many neurons in the DSCF area are responsive to rapidly-modulated call components that traverse the echo-CF_2_ range (Kanwal, 1999, 2006; Washington and Kanwal, 2008). FM selectivity is a commonality mustached bat DSCF neurons share with A1 neurons in other mammalian species (Heil et al., 1992a,b; Mendelson et al., 1993; Shamma et al., 1993; Nelken and Versnel, 2000; Zhang et al., 2003; Godey et al., 2005; Atencio et al., 2007). Further, DSCF neurons as a group show upward direction selectivity for linear FMs centered within the echo-CF_2_ range (Washington and Kanwal, 2008, 2012). Some DSCF neurons are direction selective for linear FM with durations as short as 1.3 ms and modulation rates as rapid as 4.0 kHz/ms (Washington and Kanwal, 2008). DSCF neurons are capable of responding to linear FMs with durations as short as 0.7 ms and rates as fast as 8 kHz/ms.

Even the most elaborate neural circuits are subject to physical laws. Thus, the mustached bat auditory system is no exception to the Heisenberg-Gabor Limit. The ability of DSCF neurons to detect and respond to such rapid modulations of frequency necessitates that they make use of some form of narrow temporal window. However, neurons in the DSCF area are defined by their refined spectral resolution, which requires the use of a wide temporal window. The constraint that neurons in the DSCF area must process auditory signals using both wide and narrow temporal windows creates a fundamental conflict between integration and resolution.

In theory, DSCF neurons may have evolved in such a way as to contend with this conflict. Potential strategies include (1) having one group of DSCF neurons process signals using wide temporal windows and another group using narrow windows, (2) having each neuron contain a group of synaptic or dendritic microcircuits which process signals using wide temporal windows and another set that does so using narrow windows, and (3) metabolically adjusting excitatory and inhibitory response areas such that temporal windows are wide while hunting and narrow while socializing. However, all but the first of these strategies would be energy-intensive, computationally problematic, or behaviorally untenable. The second strategy is computationally problematic on two counts. First, temporal domain computations would be performed either faster and/or at a more consistent pace than spectral domain computations, creating a bottleneck at the axon hillock by which the slow and/or intermittent flow of spectral computations interferes with the rapid and/or steady flow of temporal computations. Second, a method would be needed to differentiate between any firing patterns elicited by the spectral and temporal components of signals since they would be generated within the same neuron and thus propagating down the same axon(s). The third strategy is energy-intensive for an organism with an already high metabolism and behaviorally untenable since the bats constantly echolocate, even in social situations (Clement et al., 2006).

It is known that the same groups of neurons can accommodate multiple dimensions of stimuli within overlapping primary auditory cortex maps (e.g., cochleotopy, Merzenich et al., 1975, aural dominance, Liu and Suga, 1997, and rate selectivity, Heil et al., 1992a; Mendelson et al., 1993), much like in the primary visual cortex (e.g., retinotopy, ocular dominance, and orientation, Goodhill, 2007). However, none of the neurons constituting these overlapping maps appears to be processing stimulus dimensions derived from canonically-conjugate variables and do so on the same time scale. Although the FM-FM and DSCF areas process refined temporal and spectral information respectively, exist within the same hemisphere, and process different stimulus components, the DSCF and FM-FM areas simply do not constitute overlapping cortical maps.

The hypothesis of two functional groups of DSCF neurons, one with refined spectral resolution and another with refined temporal resolution, begs the question of how these two groups would be organized. Echolocation and Doppler Ambiguity demonstrate that canonically-conjugate variables (i.e., velocity and range via sonar) can be processed within bilateral adjacent cortical regions. However, there are key differences in the acoustic structure of biosonar signals and social calls as well as the neural circuitry used to process them. CF components always precede FM components during echolocation whereas the same cannot be said of composites (e.g., composites of the single-humped FM and short-quasi CF calls) and other sequences of social calls (Clement et al., 2006; Clement and Kanwal, 2012). As for neural circuitry, the DSCF area is located within A1 (Suga and Jen, 1976), and FM-FM is located within non-primary auditory cortex (Suga, 1985). Together, these facts illustrate the different neurocomputational constraints placed on calculating one pair of canonically-conjugate variables (velocity and range between the FM-FM and DSCF areas during echolocation) versus another (time and frequency within the DSCF area during communication). First, DSCF neurons may begin processing echo-CF components dozens of milliseconds before the FM-FM neurons even receive echo-FM information, giving them a substantial head-start in calculating velocity. Second, A1 and non-primary auditory cortex receive their own direct, separate inputs from different regions of the medial geniculate body of the thalamus (Burton and Jones, 1976; Huang and Winer, 2000). Thus, the DSCF area could start processing spectrally-based velocity information while the FM-FM area processes temporally-based range information in quasi-parallel.

Neither possibility exists for the two hypothetical populations of DSCF neurons. During communication, both neural populations would intermittently receive refined spectral information (in the form of echo-CF components of biosonar signals, CF-type syllables in the echo-CF_2_ range, or both at once) and refined temporal information (in the form of rapid FM-type syllables traversing the echo-CF_2_ range). Thus, the DSCF area would need to contend with two subpopulations that intermittently perform computations on different time scales while sharing many of the same inputs and projections. If their inputs largely originate from one cochlea (itself subject to the Heisenberg-Gabor Limit), how one population with refined spectral and another with refined temporal resolution managed to co-exist (i.e., co-evolve or co-develop) within the cochleotopic axis of A1 would be difficult to understand.

Further, any regions receiving projections from these separate populations would in all likelihood adapt, over the course of either development in the short term or evolution in the long term, by starting to specialize in spectral or temporal domain processing as well. By analogy, the neuronal coalition composing the DSCF area would be broken because its constituent neurons split into two factions that are incapable of coordinating with each other, and their conflict would eventually spread to neighboring regions.

If accurate, ASTIR would represent an elegant solution to the DSCF area's internal conflict over acoustic uncertainty. According to ASTIR, these two subpopulations of DSCF neurons could simply reside in different cerebral hemispheres. One population would be capable of slowly processing the refined spectral information necessary for tracking the velocity of a distant insect while the other population is quickly processing a steady stream of rapid FM call syllables. Communication via commissural connectivity would enable the two populations to combine information or modulate each other's activity as needed. Projections from neurons in these left and right DSCF areas to nearby cortical areas would be primarily ipsilateral, potentially resulting in entire cerebral hemispheres populated by functional areas specialized for higher-order functions ultimately rooted in temporal or spectral processing. To further stretch an analogy, ASTIR offers the spectral and temporal DSCF neural populations a most generous two-state solution.

From a population coding perspective, ASTIR holds even greater advantages over local intrahemispheric specialization for the precise processing of temporal and spectral information, especially in mustached bats. First, subregions of the auditory cortex in one hemisphere are to some degree interconnected and hierarchically organized. If a region (or set of regions) responsible for processing rapidly changing signals within a narrow temporal window is connected to an adjacent region responsible for fine frequency discrimination (a necessarily slow process relative to temporal domain processing), the resulting circuit will only be as fast as its slowest node. That is to say that the region responsible for fine frequency discrimination will become an unnecessary rate-limiting step, slowing down the processes of other adjacent regions responsible for rapid auditory processing. Housing the spectral and temporal processing regions in different hemispheres would allow the auditory cortices in both hemispheres to process signals at rates ideal for maximizing spectral and temporal information while allowing them to communicate via the corpus callosum as needed. Second, A1 is an example of primary sensory cortex. In mustached bats and other animals, there is an advantage to processing auditory signals with refined spectral (velocity) and temporal (communication) information at this level of the cortical hierarchy. Appropriating a region adjacent to a primary sensory cortical area like A1, which has different cytoarchitecture from A1 and could otherwise perform higher-level analyses on information it receives from A1, would not necessarily be evolutionarily advantageous or neuro-plastically trivial. Such a waste of cortical resources would be egregious if the sole purpose for appropriating this adjacent region was to analyze Fourier-transformed (i.e., canonically-conjugate) versions of the same auditory information processed by A1. This waste of cortical resources becomes even more nonsensical when there is another A1 on the other side of the brain that is ideally situated to perform an analysis of Fourier-transformed versions of the same information in parallel.

Taken from another perspective, ASTIR asserts that the human brain developed this specialization due to environmental pressures necessitating precise acoustic calculation of time and frequency, especially as they relate to speech and music (Zatorre et al., 2002; Poeppel, 2003). Though music's ethological purpose is still debated, its existence within every known culture suggests a role in alleviating some environmental pressures, such as adapting to living in social groups (Loersch and Arbuckle, 2013). Though spectral domain processing is necessary for detecting prosody (Lakshminarayanan et al., 2003) and speaker identity (Robin et al., 1990), humans with right auditory cortical infarct are reported as having fewer speech processing deficits than those with similar left hemispheric infarct (Purves, 2004). On the other hand, the loss of velocity tracking in a mustached bat would greatly compromise the hunting abilities of an animal with a very high metabolism, resulting in its starvation in as little as 48 h. Likewise, a mustached bat's inability to process rapid FMs, akin to receptive aphasia in a human, would likely result in social isolation, aggression from conspecifics, and/or a loss of mating opportunities. In short, the environmental pressure to develop ASTIR, the neural mechanism purported to underlie hemispheric specialization for speech and music, is arguably greater for mustached bats than for humans. Compelling environmental pressures to develop such refined spectral and temporal processing within the same auditory cortical subregion (e.g., A1) that result in an acoustic uncertainty conflict are not evident in other mammals, such as mice, cats, and macaques. The question going forward is whether such a mechanism evolved within the small brains of mustached bats and what this implies for the closed-system and domain-general hypotheses of human speech processing.

## Converge and impact: evidence and implications of hemispheric differences in mustached bat auditory cortex

Hemispheric differences in neural processing in the FM-FM area have never been the specific topic of a scientific paper. However, a prominent bat researcher reported maps of range (i.e., echo-delay) in the left and right hemispheric FM-FM areas (Suga, 1985) in a single mustached bat. This researcher concluded “that the distributions of best delays for facilitation are not the same between the left and right FM-FM areas of a single [mustached] bat.” Closer examination of these maps reveals that the left FM-FM area is highly organized and refined in the time dimension such that populations of neurons responding to fine changes in echo-delays are organized into narrow, parallel columns running along the dorsal-ventral axis. These same columns were wider and contained neurons tuned to broader echo-delays in the right hemisphere. Neurons in the left FM-FM area of this single bat have more refined temporal resolution (i.e., narrower gap detection thresholds) than those in the right FM-FM area. The sex of this bat remains unknown. Intriguing as these cortical FM-FM maps are, however, conclusions about hemispheric specialization in mustached bats cannot be extrapolated from a single animal.

On the other hand, the spectral and temporal domain processing of neurons within the left and right hemispheric DSCF areas of six bats were directly tested using linear FMs centered on each neuron's best frequency in the echo-CF_2_ range (Washington and Kanwal, 2012). Temporal domain processing was tested by varying the rates (Δ*f*/Δ*t*) of FMs, specifically by changing their durations (Δ*t*) while keeping their bandwidths (Δ*f*) constant. Spectral domain processing was tested by varying the bandwidths of FMs while maintaining their rates at the preferred rate for each neuron. FMs were always paired at onset with a CF at the best frequency in the pulse-FM_1_ range so as to optimize neural responses via facilitation.

**Figure 3:**
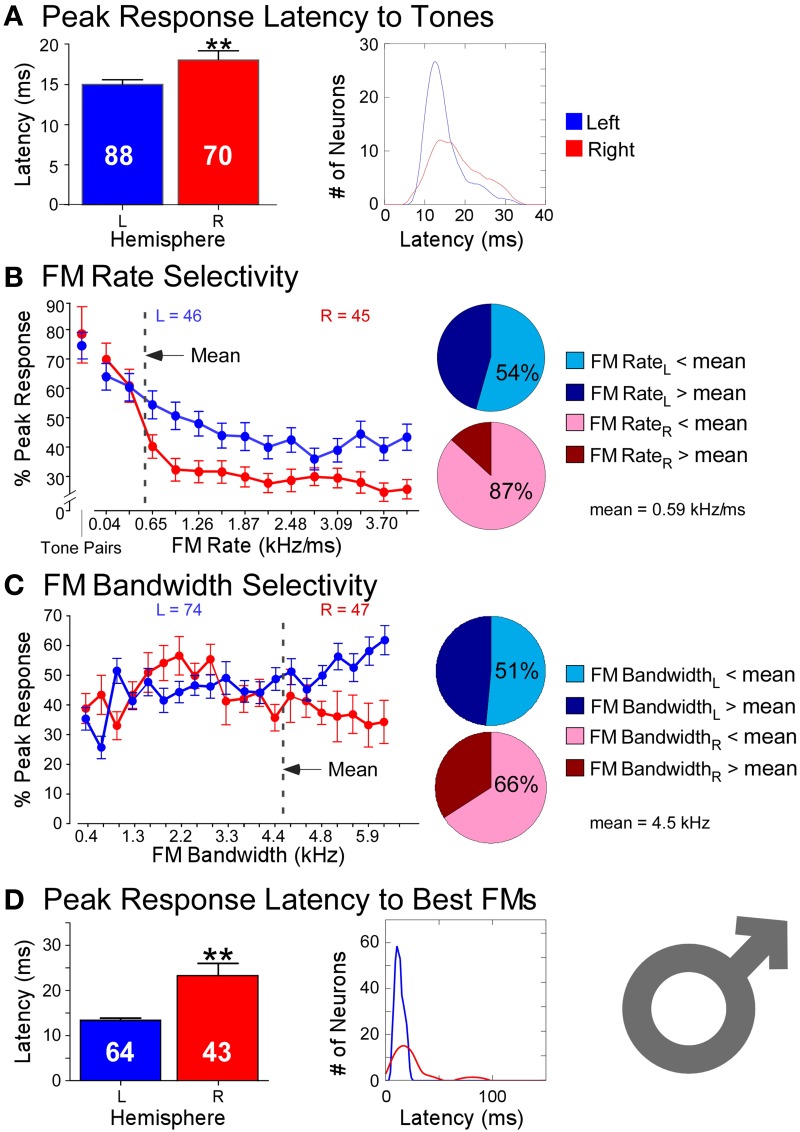
**Comparisons of temporal and spectral metrics of neural responses from left (blue) and right (red) hemispheric DSCF neurons in male mustached bats. (A)** Latencies of peak DSCF neural responses elicited by a 30-ms tone in the echo-CF_2_ range paired at onset with a 30-ms tone in the pulse-FM_1_ range. Left: Bar plot shows the average response latency for 88 left and 70 right hemispheric DSCF neurons. Right: Kernel plot shows the distribution of the same data at left. **(B)** Selectivity for the rates of FMs centered in the echo-CF_2_ range and paired at onset with a 30-ms tone in the pulse-FM_1_ range. Left: Average of normalized curves derived from magnitudes of peak DSCF neural responses (proportional to spike rates) elicited by FMs increasing in modulation rate from 0.04 to 4.0 kHz/ms in the left (46 neurons) and right (45 neurons) hemispheres. The abscissa axis shows FM rates from 0.04 to 4.0 kHz/ms and includes a separate demarcation for best tone pairs in the echo-CF_2_ and pulse-FM_1_ ranges. The ordinate axis represents the percentage of the average DSCF neuron’s peak response to FMs elicited at each rate in the 0.04–4.0 kHz/ms range. At the far left is the magnitude of the average DSCF neuron’s response to its best tone-pairs as a percentage of its maximum responses to FMs. The dotted line represents the average best FM rate of 0.59 kHz/ms. Right: Pie charts representing the percentage of left hemispheric (top) and right hemispheric (bottom) neurons with best FM rates above (dark) and below (light) the average best FM rate of 0.59 kHz/ms. **(C)** Selectivity for the bandwidths of FMs centered in the echo-CF_2_ range and paired at onset with a 30-ms tone in the pulse-FM_1_ range. Left: Average of normalized curves derived from magnitudes of peak DSCF neural responses elicited by FMs increasing in bandwidth from 0.4 to 7.9 kHz in the left (74 neurons) and right (47 neurons) hemispheres. The abscissa axis shows FM bandwidths from 0.4 to 7.9 kHz. The ordinate axis represents the percentage of the average DSCF neuron’s peak response to FMs elicited at each bandwidth in the 0.4–7.9 kHz range. The dotted line represents the average best FM bandwidth of 4.5 kHz. Right: Pie charts representing the percentage of left hemispheric (top) and right hemispheric (bottom) neurons with best FM bandwidths above (dark) and below (light) the average best FM bandwidth of 4.5 kHz. **(D)** Latencies of peak DSCF neural responses elicited by FM optimized for rate, bandwidth, and amplitude, centered in the echo-CF_2_ range, and paired at onset with a 30-ms tone in the pulse-FM_1_ range. Left: Bar plot shows the average peak response latency for 64 left and 43 right hemispheric DSCF neurons. Right: Kernel plot shows the distribution of the same data at left. Though tone-pairs generally tend to elicit greater responses in DSCF neurons than FMs, FMs optimized for rate, bandwidth, and modulation direction commonly elicit greater responses from these neurons than do tone-pairs (Washington and Kanwal, 2012). In males, the average best FM rates, durations, and bandwidths for left hemispheric DSCF neurons were 0.99 kHz/ms ± 0.13 S.E.M. (standard error of the mean), 14.04 ms ± 1.96 S.E.M., and 4.39 kHz ± 0.26 S.E.M. whereas these values for right hemispheric DSCF neurons were 0.27 kHz/ms ± 0.05 S.E.M., 34.22 ms ± 4.94 S.E.M., and 3.49 kHz kHz/ms ± 0.29 S.E.M. Thus, best FM rate and bandwidth are significantly greater (*p* < 0.05) amongst left DSCF neurons and best FM duration is greater amongst right DSCF neurons. Adapted from Washington and Kanwal (2012). Reproduced with the permission of Dr. Jagmeet S. Kanwal and the American Physiological Society.

Responses recorded from 158 neurons (*LH* = 88, *RH* = 70) in the DSCF areas of six bats in showed profound hemispheric differences that conformed to ASTIR (Figure 3). Latencies of responses elicited by pairs of CFs presented at the best frequencies in the echo-CF_2_ and pulse-FM_1_ ranges were significantly longer (*LH* = 15 ms; *RH*= 18 ms) and showed greater variance (*LH* = 21 ms; *RH* = 33 ms) amongst right DSCF neurons than those on the left. Likewise, latencies of responses elicited by FMs optimized for the spectral and temporal selectivities of each neuron (i.e., best FM bandwidths and rates) showed even greater hemispheric differences, such that latencies were nearly twice as long (*LH* = 13 ms; *RH* = 23 ms) and showed almost 20 times the variance in the right hemisphere relative to the left (*LH* = 16 ms; *RH* = 311 ms). Left DSCF neurons selected for FMs with faster rates (1 kHz/ms) than those on the right (0.2 kHz/ms) whereas right DSCF neurons selected for FMs with narrower bandwidths (3.5 kHz) than those on the left (4.4 kHz). Right DSCF neurons also selected for FMs with durations over twice as long (34 ms) as those on the left (14 ms) and had longer response durations (31 ms) than those on the left as well (20 ms). Further analyses ruled out the possibility of FM duration selectivity and hierarchical linear modeling ruled out the possibility that these results were biased to individual bats.

**Figure 4:**
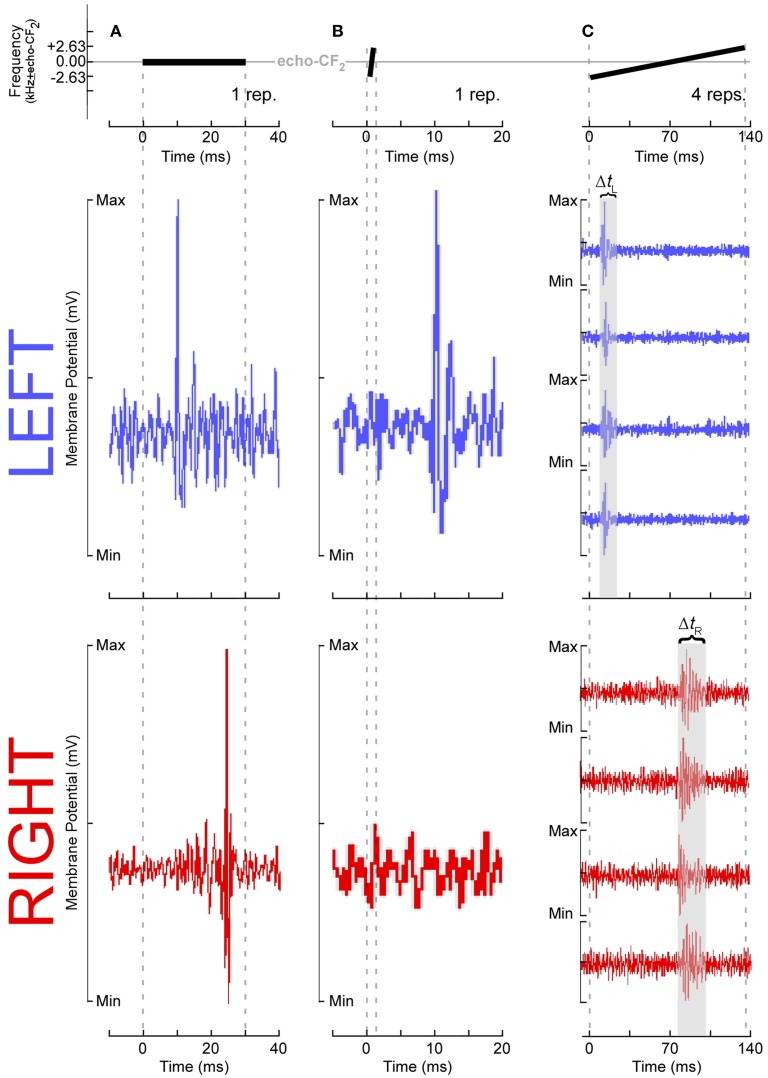
**Temporal response parameters of DSCF neurons as evidence for asymmetric sampling of time in mustached bats**. All stimuli presented in the echo-CF_2_ range (57.5–60 kHz in *P.p. rubiginosus*) were paired at onset with a 30-ms CF tone-burst in the pulse-FM_1_ (23–28 kHz). Responses shown in **(A–C)** are from six different DSCF neurons, selected because they best illustrated a particular ASTIR-related concept. (**A** top): A 30-ms, constant-frequency tone presented at echo-CF_2_. (**A** middle): Voltage trace from a typical left hemispheric DSCF neuron in a male mustached bat following one presentation of a 30-ms CF tone-burst presented at the neuron's best frequency (BF) and best amplitude of excitation (BAE). This neuron is responding within 10 ms after stimulus onset. (**A** bottom): Voltage trace from a typical right hemisphere DSCF neuron in a male mustached bat following one presentation at BAE of a 30-ms CF tone-burst centered on the neuron's BF. This neuron is responding >20 ms after stimulus onset. Left DSCF neurons typically respond to tonal stimuli 3–5 ms before those on the right in male, but not female, bats (Washington and Kanwal, 2012). Assuming DSCF neurons conform to typical integrate-and-fire models, in male moreso than female bats, ASTIR takes the form of left DSCF neurons to integrating salient stimulus features and firing in less time than right DSCF neurons. (**B** top): A 1.31-ms, upward FM centered on echo-CF_2_, which has a modulation rate of 4 kHz/ms and a bandwidth of 5.25 kHz. (**B** middle): Voltage trace from a typical left DSCF neuron in a male bat following one presentation of a 5.25 kHz, 4 kHz/ms upward FM at BAE and centered on the neuron's BF. This neuron is responding within 10 ms after stimulus onset. (**B** bottom): Voltage trace from a typical right DSCF neuron in a male bat following presentation at BAE of a 5.25 kHz, 4 kHz/ms upward FM centered at the neuron's BF. This neuron is simply not responding. Relative to left DSCF neurons, right DSCF neurons are less responsive to shorter FM signals (Washington and Kanwal, 2012). This selectivity for longer sounds suggests right DSCF neurons have longer integration windows and are thus less likely to respond to such short sounds. Though this hemispheric difference is observed in both sexes, it is more pronounced in males. (**C** top): A 131-ms, upward FM centered at echo-CF_2_, which has a modulation rate of 0.04 kHz/ms and a bandwidth of 5.25 kHz. (**C** middle): Voltage traces from a typical left DSCF neuron in a male bat following four presentations at BAE of a 5.25 kHz, 0.04 kHz/ms upward FM centered on the neuron's BF. This neuron's four responses are time-locked and occur within the first 30 ms of the stimulus. (**C** bottom): Voltage traces from a typical right DSCF neuron in a male bat following four presentations at BAE of a 5.25 kHz, 0.04 kHz/ms upward FM centered at the neuron's BF. This neuron's four responses are not time-locked (i.e., tonic or burst firing) and occur after the first 70 ms of the stimulus. In both sexes, the maximum response duration of the left DSCF neuron (Δ*t*_L_) is less than that of the right DSCF neuron (Δ*t*_R_) (Washington and Kanwal, 2012). Since, in general, Δ*t*_R_ > Δ*t*_L_, right DSCF neurons in general are less capable of processing precise temporal information than left DSCF neurons. Washington and Kanwal, unpublished data, reproduced with the permission of Jagmeet S. Kanwal, PhD.

These results require some further explanation. Left DSCF neurons had generally less selectivity than those on the right. For instance, although left DSCF neurons selected for faster FM rates, they were more likely to respond to a multitude of FM rates, both rapid and slow. These FM rate selectivity results are consistent with behavioral (Schwartz and Tallal, 1980) and neuroimaging (Belin et al., 1998) results for formant transitions in humans. Their responses to FM bandwidths could be similarly characterized. Right DSCF neurons on the other hand generally responded robustly to long, slow, narrowband FMs but showed few if any responses to short, rapid, or broadband FMs. Indeed, on average, there is a 900 Hz difference (LH > RH) in best FM bandwidth between left and right DSCF neurons, which is 18 times the spectral resolution the bat needs to detect differences between a pulse- and echo-CF_2_ (Riquimaroux et al., 1992; Washington and Kanwal, 2012). Their selectivity for longer, narrowband sounds and their longer response durations strongly suggest that, consistent with ASTIR, right hemispheric DSCF neurons employ longer temporal integration windows relative to those on the left. Further, left and right DSCF neurons differ not only in their ability to detect rapid changes in stimulus features but also they differ in how quickly and for how long they respond to stimuli in general. FM rate selectivity and response latency would appear to be unrelated measures, but they both reflect finer temporal domain processing in left hemispheric DSCF neurons relative to those on the right. Right hemispheric DSCF neurons take longer to respond to stimuli and have less reliable spike times (i.e., less time-locked) than their left hemispheric counterparts. The finding that multiple temporal measures (i.e., FM rate selectivity, latency, response duration, etc.) are shorter and/or more refined in the left hemisphere suggests that hemispheric differences in the widths of temporal integration windows manifests in multiple ways, even at the single neuron level, in the mustached bat's A1 (Figure 4).

There is one factor mitigating the results above. The results represent only half of the mustached bat population: Males.

**Figure 5:**
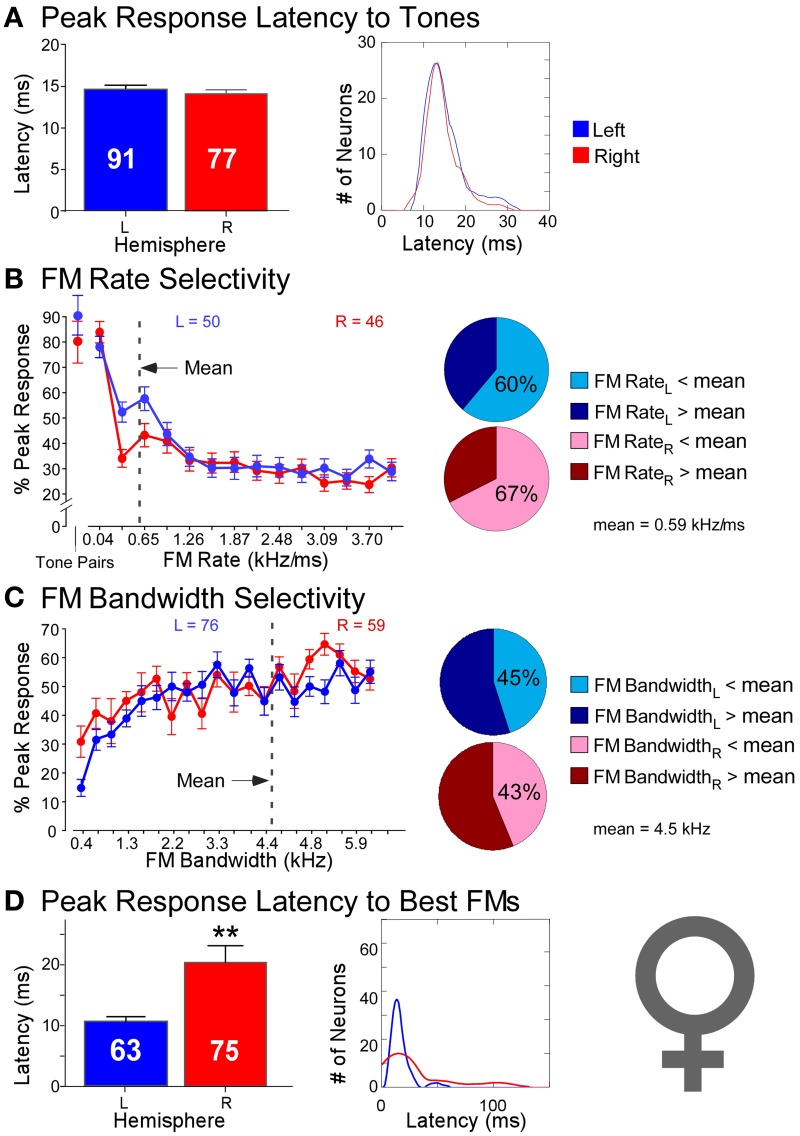
**Comparisons of temporal and spectral metrics of neural responses from left (blue) and right (red) hemispheric DSCF neurons in female mustached bats. (A)** Latencies of peak DSCF neural responses elicited by a 30-ms tone in the echo-CF_2_ range paired at onset with a 30-ms tone in the pulse-FM_1_ range. Left: Bar plot shows the average response latency for 91 left and 77 right hemispheric DSCF neurons. Right: Kernel plot shows the distribution of the same data at left. **(B)** Selectivity for the rates of FMs centered in the echo-CF_2_ range and paired at onset with a 30-ms tone in the pulse-FM_1_ range. Left: Average of normalized curves derived from magnitudes of peak DSCF neural responses elicited by FMs increasing in modulation rate from 0.04 to 4.0 kHz/ms in the left (50 neurons) and right (46 neurons) hemispheres. The abscissa axis shows FM rates from 0.04 to 4.0 kHz/ms and includes a separate demarcation for best tone pairs in the echo-CF_2_ and pulse-FM_1_ ranges. The ordinate axis represents the percentage of the average DSCF neuron's peak response to FMs that is elicited at each rate in the 0.04–4.0 kHz/ms range. At the far left is the magnitude of the average DSCF neuron's response to its best tone-pairs as a percentage of its maximum responses to FMs. The dotted line represents the average best FM rate of 0.59 kHz/ms. Right: Pie charts representing the percentage of left hemispheric (top) and right hemispheric (bottom) neurons with best FM rates above (dark) and below (light) the average best FM rate of 0.59 kHz/ms. **(C)** Selectivity for the bandwidths of FMs centered in the echo-CF_2_ range and paired at onset with a 30-ms tone in the pulse-FM_1_ range. Left: Average of normalized curves derived from magnitudes of peak DSCF neural responses elicited by FMs increasing in bandwidth from 0.4 to 7.9 kHz in the left (76 neurons) and right (59 neurons) hemispheres. The abscissa axis shows FM bandwidths from 0.4 to 7.9 kHz. The ordinate axis represents the percentage of the average DSCF neuron's peak response to FMs that is elicited at each bandwidth in the 0.4–7.9 kHz range. The dotted line represents the average best FM bandwidth of 4.5 kHz. Right: Pie charts representing the percentage of left hemispheric (top) and right hemispheric (bottom) neurons with best FM bandwidths above (dark) and below (light) the average best FM bandwidth of 4.5 kHz. **(D)** Latencies of peak DSCF neural responses elicited by FM optimized for rate, bandwidth, and amplitude, centered in the echo-CF_2_ range, and paired at onset with a 30-ms tone in the pulse-FM_1_ range. Left: Bar plot shows the average peak response latency for 63 left and 75 right hemispheric DSCF neurons. Right: Kernel plot shows the distribution of the same data at left. Adapted from Washington and Kanwal (2012). In females, the average best FM rates, durations, and bandwidths for left hemispheric DSCF neurons were 0.41 kHz/ms ± 0.06 S.E.M. (standard error of the mean), 36.68 ms ± 5.65 S.E.M., and 4.93 kHz ± 0.29 S.E.M. whereas these values for right hemispheric DSCF neurons were 0.44 kHz/ms ± 0.10 S.E.M., 62.32 ms ± 8.69 S.E.M., and 4.60 kHz kHz/ms ± 0.32 S.E.M. Thus, only best FM duration is significantly different such that it is greater amongst right DSCF neurons. Adapted from Washington and Kanwal (2012). Reproduced with the permission of Dr. Jagmeet S. Kanwal and the American Physiological Society.

The same study described above reported recordings not only from neurons in the DSCF areas of six male mustached bats but also reported recordings from 168 neurons (*LH* = 91, *RH* = 77) in the DSCF areas of four female mustached bats (Washington and Kanwal, 2012). While sometimes significant, hemispheric differences in spectral versus temporal processing were decidedly less pronounced in females than in males (Figure 5). Latencies of responses to CF-pairs were remarkably similar in duration (*LH* = 15 ms; *RH* = 14 ms) and variance (*LH* = 15 ms; *RH* = 20 ms) across hemispheres, comparable to those of the left hemisphere in males. Left and right DSCF neurons also selected for FMs with similar rates (*LH* = 0.41 kHz/ms; *RH* = 0.47 kHz/ms) and bandwidths (*LH* = 4.93 kHz; *RH* = 4.74 kHz). However, like males, right DSCF neurons from female bats also selected for FMs with significantly longer durations (65 ms) and had longer response durations relative to those on the left (37 ms). Likewise, response latencies to FMs in females were significantly longer on the right (30 ms) than on the left (17 ms), similar to males. Left DSCF neurons were again less selective for slow FM rates in females but to a far lesser extent than in males. There were no appreciable hemispheric differences in spectral domain processing in females. Yet, response characteristics of neurons in the right DSCF areas of female bats showed multiple signs of processing sounds using longer integration windows relative to those in the left hemisphere. Like in males, female bats displayed no selectivity for FM durations, and these results were not biased to individual bats.

Despite these sex differences in hemispheric specialization, what must be emphasized is that ASTIR appears to be a feature of both the male and female mustached bat auditory cortex. Further, data collected from multiple species suggests that these sex differences represent less a flaw in the hypothesis proposed here than a feature of hemispheric specialization for communication sounds. Hemispheric specialization for song production and perception is greater in male than in female songbirds (Nottebohm and Arnold, 1976; DeVoogd and Nottebohm, 1981). Male songbirds are also able to use both spectral and temporal information to classify call stimuli by the sex of the caller but females can only use temporal information (Vicario, 2004). Certainly, hemispheric specialization for speech and language is often, but not always (Obleser et al., 2001, 2004), found to be stronger in men than in women (Lansdell, 1964; McGlone, 1977; Dawe and Corballis, 1986; Shaywitz et al., 1995). Men are reported to have greater left hemispheric specialization (i.e., right-ear-advantage) for temporal domain processing than women as well (Brown et al., 1999). A sex-dependent asymmetry in mustached bat auditory cortex implies that this asymmetry is at least analogous to the asymmetries found in songbirds, rats, and humans. Please note that, though there was no evidence for right-lateralized refined spectral domain processing in female bats, refined spectral processing in the right hemispheric DSCF areas of male bats was less statistically robust than refined temporal processing in the left hemisphere. Thus, the apparent lack of right-lateralized, refined spectral domain processing in female bats may simply reflect their overall diminished hemispheric specialization relative to males.

Placing mustached bat echolocation and communication into a computational context via the Heisenberg-Gabor Limit allows us to begin answering longstanding questions. First, the fact that ASTIR appears to be greater in male bats and that advantages for temporal and spectral domain processing are found in the left and right hemispheres respectively, and not vice-versa, strongly suggest that hemispheric specialization in mustached bats is analogous to such specialization in the human brain. Second, ASTIR's presence within the brains of mustached bats, when coupled with the fact that neither echolocation nor communication represents a closed system in this species, is evidence against closed-system hypotheses of speech processing. If ASTIR can occur in mustached bats and amongst the same neurons responsible for processing both biosonar signals and social calls, a language-only “speech organ” existing within the left superior temporal gyri of humans seems unnecessary. Third, ASTIR's presence in mustached bats is even stronger evidence against brain-volume and handedness hypotheses. The small brains of mustached bats and the large brains of humans are capable of having similar hemispheric differences. Mustached bats also do not have hands or even use tools.

Nonetheless, this theoretical discussion of neural mechanisms of hemispheric specialization and the evidence supporting their existence in mustached bat auditory cortex raises many questions. Those questions stemming from sex differences are admittedly some of the most difficult: Why do these sex differences exist? What adaptive purpose do they serve? The hypothesis presented above asserts that powerful ethological pressures related to hunting and socialization in mustached bats underlies the development of ASTIR in mustached bat auditory cortex. However, some form of ethological pressure also drove hemispheric specialization for communication in humans and songbirds while leaving some startling exemptions for females in those species. It is likely that the sex differences for hemispheric specialization in mustached bats are present for the same reason similar sex differences are present in humans and songbirds. However, there is no consensus on why these sex differences exist in any of these species. Testosterone levels *in-utero* and during infancy are known to modulate hemispheric specialization for speech and language in humans (Geschwind and Galaburda, 1985; Tallal et al., 1988, 1993; Beech and Beauvois, 2006). However, such mechanistic explanations do not adequately address the ethological question as to why such sex differences evolved in the first place.

To this end, current results in the mustached bat may be more useful for questioning answers than for answering questions. Specifically, anthropologists have associated sex differences in hemispheric specialization for speech and language with the respective hunter and gatherer roles of men and women (Joseph, 2000). This anthropological explanation is inadequate for mustached bats since the males and females in this species are both insectivorous hunters. There are behavioral differences between male and female mustached bats relevant to how they process both biosonar signals and social calls: pulse-CFs_2_ are higher in frequency amongst female bats (Suga et al., 1987), males emit social calls more often than females, and the sexes differ in the types of social calls they emit (Clement and Kanwal, 2012). Pharmacobehavioral techniques previously used to determine DSCF frequency resolution (Riquimaroux et al., 1992) could be altered (e.g., unilateral muscimol application) so as to determine the extent to which the left and right hemispheric DSCF areas differ in spectral resolution in male and female mustached bats. Further, field studies of mustached bats that ask behavioral questions framed by comparisons between sexes (e.g., is there finer-tuned velocity tracking amongst males?) would shed light on the reasons for this phenomenon in humans and birds. Such field studies could ultimately have a surprising impact on anthropological theories concerning the evolution of hemispheric specialization for speech and language.

Neurophysiological studies employing more sophisticated equipment and experimental designs will be needed to fully explore this sex dependent asymmetry. Mapping studies could determine if males and females have a different spatial distribution of neurons in the auditory cortex or a different morphology of auditory cortical fields. Neuropharmacological techniques previously used in the study of neural selectivity for social calls in bats (Klug et al., 2002) could be employed to manipulate GABA, glutamate, and perhaps even sex hormones to observe how they alter the firing of left and right hemispheric neurons in the auditory cortices of male and female mustached bats. Further, the evidence presented above suggests that right hemispheric DSCF neurons in males would be more selective for CF-type social calls relative to left hemispheric DSCF areas in male bats or either hemisphere in female bats. An otherwise rigorous study of hemispheric differences in the processing of social calls in the DSCF area did not address this key question (Kanwal, 2012). Neuroimaging could not only determine this sex difference's consistency across animals but also would determine the extent to which hemispheric specialization for audiovocal communication in general pervades the mustached bat auditory cortex, much like neuroimaging studies have in songbird nuclei (Poirier et al., 2009). Far from being a simple scientific anomaly, sex-dependent ASTIR in mustached bats may inspire experiments that will unravel persistent neurophysiological, phonological, and anthropological mysteries.

### Conflict of interest statement

The authors declare that the research was conducted in the absence of any commercial or financial relationships that could be construed as a potential conflict of interest.
