# Supplementary material for: Conjugating time and frequency: hemispheric specialization, acoustic uncertainty, and the mustached bat
Source: Front Neurosci. 2015 Apr 27;9:143. doi: 10.3389/fnins.2015.00143 (PMC4410141; doi:10.3389/fnins.2015.00143)
Supplement: Supplementary file 1 [file SupplementarySection1.DOCX]

Supplementary Section 1. The Acoustic Uncertainty Principle

The Acoustic Uncertainty Principle states that frequency and time are canonically-conjugate variables of sound such that increasing the precision of frequency-related measurements (i.e., spectral resolution) for sounds must decrease the precision of time measurements (i.e., temporal resolution) of those same sounds and vice-versa [1]. The Acoustic Uncertainty Principle is a direct consequence of the same mathematics underlying Heisenberg’s well-known Quantum Uncertainty Principle, which states that the variables of *momentum* and *position* are likewise canonically-conjugate when applied to electrons. Electrons and other physical quanta can be modelled as waves and thus expressed as combinations of frequencies. Similarly, sounds are expressed as waves and frequencies.

A sound wave’s amplitude at time *t* is given by a function *A(t)*. The function *A(t)* can be decomposed as a combination of single-frequency waves $e^{ift}$ for all frequencies *f*:

$$A(t)=\frac{1}{\sqrt{2\pi}} \int_{-\infty}^{\infty} \hat{A}\left( f \right) e^{ift}df$$

where $\hat{A}(f)$ is the Fourier transform of *A(t)*.

The wave energy that arrives per unit time is proportional to *A(t)^2^*, and the total energy of the wave is $E=\int{A(t)}^{2}dt$. Likewise, the energy carried by each frequency *f* is $\left| \hat{A}(f) \right|^{2}$. $\int\left| \hat{A}(f) \right|^{2}df is also equal to E$.

An incoming sound wave has both a *mean arrival time* $\bar{t}$ and a *mean frequency* $\bar{f}$ , but it always has a finite duration and frequency distribution. The temporal resolution ($\Delta t$) and spectral resolution ($\Delta f$) are defined as the standard deviations of *t* and *f* when weighted by the energy intensity:

$${\Delta t}^{2}=\frac{1}{E}\int{{A(t)}^{2}\left( t-\bar{t} \right)}^{2}dt {\Delta f}^{2}=\frac{1}{E}\int\left| \hat{A}\left( f \right) \right|^{2}\left( f-\bar{f} \right)^{2}df$$

Heisenberg’s inequality [2] states that for any *A(t)*, the product of these two integrals has a lower bound equal to 1/${16\pi}^{2}$. Thus,

$${\Delta t}^{2}{\cdot\Delta f}^{2}\geq\frac{1}{{16\pi}^{2}}$$

which can be simplified into the standard form of the Heisenberg-Gabor Limit:

$$\Delta t \cdot\Delta f \geq\frac{1}{4\pi}$$

The Heisenberg-Gabor Limit is the mathematical basis of the Acoustic Uncertainty Principle. Spectrograms of the spoken NU-6 List 1A word “dime” (Main Text Figure 1) illustrate the inverse relationship between temporal resolution ($\Delta t$) and spectral resolution ($\Delta f$) as expressed within the Heisenberg-Gabor Limit. Using a wide temporal window for Fourier transforms yields a spectrogram with (a) high spectral resolution due to there being many independent frequency channels and (b) low temporal resolution due to the merging (i.e., “blurring”) of successive, rapid acoustic events into the wide window. Conversely, a Fourier transformation implementing a narrow temporal window yields a spectrogram with (a) high temporal resolution due to the segregation of successive, rapid acoustic events and (b) low spectral resolution due to the merging of harmonics into a small number of independent frequency channels.

Please note that the Heisenberg-Gabor Limit further states that even a system employing both narrow and wide temporal windows would fail to simultaneously obtain refined temporal and spectral information, even when both windows are analysing the same function. Nevertheless, a system resigned to measuring temporal and spectral information on different time-scales (i.e., sacrificing the *simultaneity* constraint) may still acquire precise temporal and spectral information from the same signal. That is to say, acquisition of refined spectral information in such a multi-window system would be inevitably slower and/or temporally inconsistent relative to the acquisition of refined temporal information.

Asymmetry for Spectral versus Temporal Integration and Resolution (ASTIR) is a processing strategy theorized to have evolved within the brains of humans (and other species, potentially) to account for the spectral-temporal trade-off central to acoustic uncertainty. ASTIR states that the left auditory cortex has superior temporal domain processing (e.g., temporal resolution) and the right has superior spectral domain processing (e.g., spectral resolution). ASTIR suggests that hemispheric specializations for speech and music within the left and right auditory cortices, respectively, are secondary indications that the human auditory system has evolved to process sounds using both high temporal and high spectral resolution.

The terminology used here and throughout phonological literature is worthy of discussion.  A 2003 paper by Poeppel introduced the term Asymmetric Sampling in Time (AST).  The AST paper is often described as promoting the idea that hemispheric differences in the widths of acoustic temporal integration windows underlie left hemispheric specialization for speech.  The ideas in Poeppel’s AST paper are often contrasted with those in Zatorre, Belin, and Penhume’s 2002 paper [3].  Zatorre, Belin, and Penhume state that it is advantageous for one hemisphere to process refined spectral information and for the other hemisphere to process refined temporal information, giving rise to hemispheric specialization for speech and music [4].  Closer examination of these papers shows that the hypotheses they espouse are mathematically equivalent. Poeppel’s AST paper (pp. 249-250) states that these proposed hemispheric differences in the widths of temporal integration windows would lead to hemispheric differences in spectral and temporal resolution.  Likewise, Zatorre, Belin, and Penhume’s “acoustic uncertainty” paper (pg. 41, Box 2) states that the size of the temporal integration window is critical to the spectral and/or temporal resolution necessary for music and speech perception.  The tradeoff between spectral and temporal resolution is given by the Heisenberg-Gabor Limit. The papers do differ on the role of A1. Otherwise, these hypotheses appear to be examples of “multiple independent discovery” in science and mathematics and should be credited to both groups. The term introduced here, ASTIR, is an umbrella term encompassing AST and “acoustic uncertainty.” It is the authors’ viewpoint that ASTIR should be considered equivalent to AST and “acoustic uncertainty” just as these terms should be considered equivalent to each other.

[1] Joos, M. 1948 *Acoustic Phonetics*, Lingusitic Society of America.

[2] Wiener, N. 1933 *The Fourier integral and certain of its applications*. Cambridge [Eng.], The University press; xi, 201, [201] p. p.

[3] Poeppel, D. 2003 The analysis of speech in different temporal integration windows: cerebral lateralization as ‘asymmetric sampling in time’. *Speech Communication* 245–255.

[4] Zatorre, R.J., Belin, P. & Penhune, V.B. 2002 Structure and function of auditory cortex: music and speech. *Trends Cogn Sci* **6**, 37-46.
