# Supplementary material for: Conjugating time and frequency: hemispheric specialization, acoustic uncertainty, and the mustached bat
Source: Front Neurosci. 2015 Apr 27;9:143. doi: 10.3389/fnins.2015.00143 (PMC4410141; doi:10.3389/fnins.2015.00143)
Supplement: Supplementary file 2 [file SupplementarySection2.DOCX]

Supplementary Section 2. Spectral and temporal processing in the DSCF area

The Doppler-shifted constant frequency (DSCF) processing area occupies the central-most 50% of primary auditory cortex in mustached bats [1] and serves a role in both echolocation and communication [2]. We hypothesize that the processing of biosonar signals and social calls by DSCF neurons employs computational strategies for processing the canonically-conjugate variables of frequency and time, which drive hemispheric specialization for spectral and temporal domain processing.

Specifically, neurons in the DSCF area calculate target velocity by measuring minute frequency differences between the pulse-CF_2_ (a frequency to which the DSCF neurons are mostly unresponsive) and the returning, Doppler-shifted echo-CF_2_ (a frequency to which the majority of DSCF neurons are most responsive) [1]. DSCF neurons evolved to be unresponsive to any echo-CFs_2_ that are Doppler-shifted downward in frequency relative to the pulse since such frequency shifts would have one of two improbable implications: (1) the prey insect is flying faster than a pursuing bat or (2) the bat is flying backward relative to the prey insect. Thus, echo-CFs_2_ must be Doppler-shifted upward in frequency (e.g., approaching an insect) to excite DSCF neurons, and each neuron has selective tuning for a particular echo-CF_2_ (i.e., a particular velocity). Detecting such minute differences in frequency requires refined spectral resolution, which can only be achieved through the sacrifice of refined temporal resolution per acoustic uncertainty.

Subtle behaviors exhibited by the mustached bat as its DSCF neurons calculate target velocity reveal how critical acoustic uncertainty is to echolocation [3]. Once a mustached bat detects a prey insect, it lengthens CF_1-4_ from roughly 17 to 34 ms while emitting biosonar pulses at the low rate of 5/sec. Lengthening CF_1-4_ while emitting biosonar pulses at a slow rate effectively narrows the maximum spectral window and allows the bat to perform more accurate velocity calculations. As the bat nears its insect prey, however, the distance (i.e., the maximum temporal window) between them becomes narrower, reducing the maximum spectral resolution. This monotonic decrease in the bat’s ability to precisely track target velocity becomes increasingly irrelevant to the pursuit as the bat begins to match the insect’s velocity. During this time, the bat also increases pulse-rate (up to 100/sec) and systematically shortens CF_1-4_ thus increasing temporal resolution and better enabling the bat to track the insect’s range. Neurons in the FM-FM processing area gradually become more responsible for processing the primarily range-related information carried by these shortened, predominantly frequency-modulated biosonar signals whereas the role of DSCF neurons gradually diminishes.

DSCF neurons also process FMs present within social calls [4]. DSCF neurons are capable of responding selectively to FM features within social calls that are as short as 1 ms, have FM rates as rapid as 4 kHz/ms, and traverse the echo-CF_2_ range. Thus, environmental pressures dictate that DSCF neurons are required to precisely calculate two canonically conjugate variables, a mathematical impossibility. ASTIR states that humans devised a computational strategy to satisfy similar environmental demands by specializing the left auditory cortex for temporal domain processing and the right for spectral domain processing [5, 6]. Since bats, like humans and other mammals, are bilateral, they have two DSCF areas (one in the left hemisphere and the other in the right). One of these DSCF areas (e.g., right) could have sharper spectral resolution and the other (e.g., left) sharper temporal resolution, enabling the bat to calculate velocity via echolocation, to process social calls, and to do both with precision.

[1] Suga, N. & Jen, P.H. 1976 Disproportionate tonotopic representation for processing CF-FM sonar signals in the mustache bat auditory cortex. *Science* **194**, 542-544.

[2] Kanwal, J.S. 1999 Processing species-specific calls by combination-sensitive neurons in an echolocating bat. In *The Design of Animal Communication* (eds. M.D. Hauser & M. Konishi), pp. 135-157. Cambridge, MA, The MIT Press.

[3] Suga, N. 1985 The extent to which biosonar information is represented in the bat auditory cortex. In *Neurocomputing 2: Directions for Research* (eds. J.A. Anderson, A. Pellionisz & E. Rosenfeld), pp. 259-294. Cambridge, MA, MIT Press.

[4] Washington, S.D. & Kanwal, J.S. 2008 DSCF neurons within the primary auditory cortex of the mustached bat process frequency modulations present within social calls. *J Neurophysiol* **100**, 3285-3304.

[5] Zatorre, R.J., Belin, P. & Penhune, V.B. 2002 Structure and function of auditory cortex: music and speech. *Trends Cogn Sci* **6**, 37-46.

[6] Poeppel, D. 2003 The analysis of speech in different temporal integration windows: cerebral lateralization as ‘asymmetric sampling in time’. *Speech Communication* 245–255.


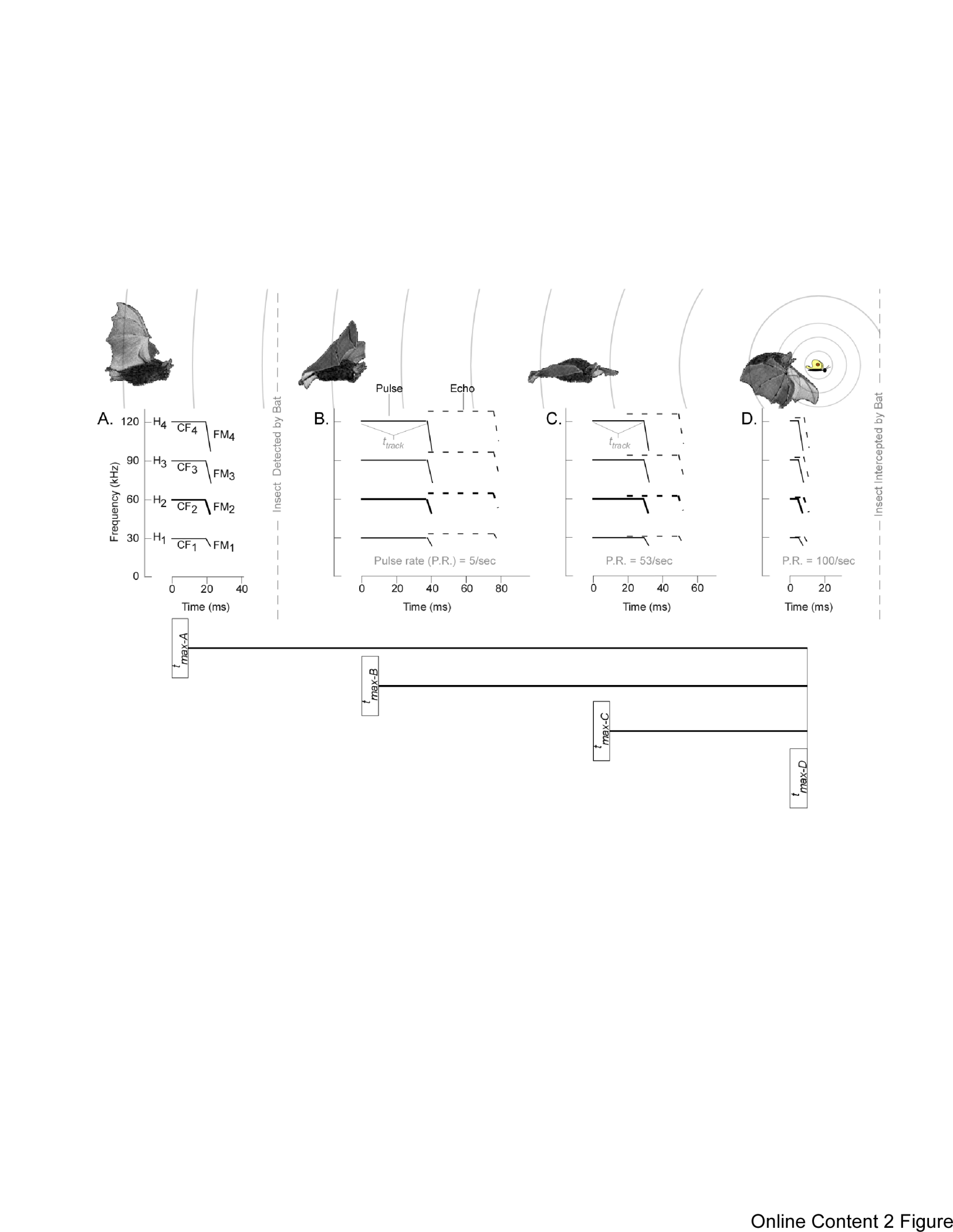


**Online Content 2 FIGURE LEGEND**

Online Content 2 Figure. Acoustic uncertainty during hunting behavior. (Top) A mustached bat flies toward a prey insect (moth) whose wing-beats emit sounds that the bat can track. (Middle) Biosonar signals (H_1-4_) emitted by the mustached bat during echolocation. These sonar signals may be further broken down into CF (CF_1-4_) and FM (FM_1-4_) components used to track velocity and range, respectively. Solid-line signals represent biosonar *pulses*, which are emitted by the bat, and hatched-line signals represent *echoes* returning from the target. Note that when a bat flies toward a “fluttering” target, such as a moth, the Doppler-shifted of the echo is composed of both a steady shift in frequency proportional to the relative velocity and a periodic FM proportional to the speed of the moth’s wing beat. For simplicity, we consider only the steady-shift in this discussion. (Bottom) The distance between the tip of the bat’s nose and the moth is the maximum temporal window *t_max_*. Since the bat gets progressively closer to the moth in examples A-D, *t_max_* diminishes in each example as well such that *t_max-A_* > *t_max-B_* > *t_max-c_* > *t_max-D_*. The duration of the CF component represents the actual tracking temporal window *t_track_*. A. The mustached bat emits sonar pulses into the environment prior to detecting the moth. The absence of echo-H_1-4_ in this depiction is not meant to suggest that the bat is no receiving echo information from the environment, but rather that it is receiving no such information from the moth. B. Once the bat is close enough to the moth to detect it, the bat extends its CF components from 17 to 34 ms. Per the Heisenberg-Gabor Limit, this elongation of the CF component (*t_track_*) enhances the bat’s ability to track the moth’s velocity. The FM components remain 3 ms in duration and the bat is delivering pulses at the slow rate of 5/sec. C. As *t_max_* decreases, *t_track_* is shortened due to bat nearly matching the moth’s velocity and the diminished precision of the velocity information that the bat is receiving. The narrower *t_max_* becomes, the poorer the velocity information the bat is theoretically capable of receiving. The bat has increased its pulse rate to over 50/sec. D. The bat has matched the velocity and largely closed the distance between itself and the moth. Having only a very narrow *t_max_* that it can still use to obtain any additional velocity information, the bat has decreased the duration of its pulse-CF components (*t_track_*) and increased its pulse rate to 100/sec. Such velocity information is of little use at this stage of the pursuit. The bat ultimately intercepts (and presumably eats) the moth.
